# 3D-StyleGAN2-ADA: Volumetric Synthesis of Realistic Prostate T2W MRI

**DOI:** 10.3390/jimaging12030130

**Published:** 2026-03-14

**Authors:** Claudia Giardina, Verónica Vilaplana

**Affiliations:** Signal Theory and Communications, Universitat Politècnica de Catalunya—BarcelonaTech (UPC), 08034 Barcelona, Spain; claudia.giardina@upc.edu

**Keywords:** prostate MRI, generative adversarial network, 3D-StyleGAN2-ADA, medical image synthesis, prostate cancer, MRI augmentation

## Abstract

This work investigates the extension of StyleGAN2-ADA to three-dimensional prostate T2-weighted (T2W) MRI generation. The architecture is adapted to operate on 3D anisotropic volumes, enabling stable training at a clinically relevant resolution of 256×256×24, where a baseline 3D-StyleGAN fails to converge. Quantitative evaluation using Fréchet Inception Distance (FID), Kernel Inception Distance (KID), and generative Precision–Recall metrics demonstrates substantial improvements over a 3D-StyleGAN baseline. Specifically, FID decreased from 114.2 to 27.3, while generative Precision increased from 0.22 to 0.82, indicating markedly improved fidelity and alignment with the real data distribution. Beyond generative metrics, the synthetic volumes were evaluated through radiomic feature analysis and downstream prostate segmentation. Synthetic data augmentation resulted in segmentation performance comparable to real-data training, supporting that volumetric generation preserves anatomically relevant structures, while multivariate radiomic analyses showed strong global feature alignment between real and synthetic volumes. These findings indicate that a 3D extension of StyleGAN2-ADA enables stable high-resolution volumetric prostate MRI synthesis while preserving anatomically coherent structure and global radiomic characteristics.

## 1. Introduction

Prostate cancer is one of the most common malignancies in men worldwide and a leading cause of cancer-related mortality [1]. Early diagnosis and accurate staging are essential for effective treatment and patient management. Among imaging modalities, prostate magnetic resonance imaging (MRI), especially the T2-weighted (T2W) sequence, has become a cornerstone in prostate cancer diagnosis, thanks to its superior soft-tissue contrast. T2W imaging is particularly effective for delineating the prostate gland, its internal zonal anatomy, and surrounding tissues, facilitating the detection of lesions indicative of clinically significant prostate cancer (csPCa) [2,3,4]. As such, it plays a central role in prostate MRI protocols, including biparametric MRI (bpMRI).

Despite its diagnostic value, MRI interpretation is inherently complex, requiring expert radiologists with specific training in prostate imaging. Furthermore, it is time-consuming and labor-intensive, as it involves reviewing numerous images per patient [5].

To support radiologists and reduce interpretation burden, machine learning (ML) methods have been developed to assist with lesion detection and classification. However, the success of ML models depends on access to large, diverse, and well-annotated datasets. In prostate MRI, creating such datasets is particularly difficult, as it demands expert delineation of anatomical structures and lesions, a time-intensive and costly process. This limitation continues to hinder the development and generalization of robust and transferable ML solutions [6].

Generative models, particularly Generative Adversarial Networks (GANs) [7], offer a promising solution for synthesizing realistic medical images to augment training datasets. In medical imaging, GANs have been applied to data augmentation, domain adaptation, and anomaly detection [8,9,10,11]. In prostate imaging, 2D GAN-based methods have been used to augment training data and improve downstream tasks such as segmentation and classification [12,13]. However, these 2D approaches are limited in their ability to capture volumetric anatomical continuity, which is crucial for realistic MRI generation and clinical applicability.

In prior experiments on 2D prostate MRI synthesis, StyleGAN2-ADA and latent diffusion models (LDMs) were compared to assess slice-wise generation performance. StyleGAN2-ADA achieved higher fidelity and substantially faster training and inference than LDMs [14], establishing it as a stable and efficient backbone for medical image generation. These findings motivated its extension to volumetric data to overcome the spatial limitations of 2D models.

Building on this foundation and motivated by the need for 3D prostate MRI synthesis, the present work extends StyleGAN2-ADA to operate directly on 3D data for high-resolution T2W prostate MRI synthesis. The study further investigates whether volumetric generation preserves anatomical structure, aligns with the real data distribution, and provides practical insights in downstream analysis.

The main contributions are as follows:

- Extension of the StyleGAN2-ADA architecture to three-dimensional volumetric synthesis, enabling stable training at clinically relevant resolution.
- Comprehensive evaluation of synthetic image realism using generative fidelity metrics, radiomic feature analysis, and downstream prostate segmentation experiments.

The remainder of this paper is organized as follows. Section 2 reviews the existing literature on prostate MRI synthesis and GAN-based methods in the medical domain. Section 3 describes the adaptation of StyleGAN2-ADA to 3D, training setup, and evaluation protocols. Section 4 presents the experimental results, covering fidelity and diversity metrics, and downstream evaluation through segmentation and radiomics. Section 5 discusses the main findings, strengths, and limitations of the proposed approach, and outlines directions for future work. Finally, Section 6 summarizes the conclusions.

## 2. Related Work

Generative models play a key role in medical image synthesis, supporting applications such as data augmentation, domain translation, and anomaly detection. This section reviews recent advances in 3D medical image generation using GANs, with emphasis on methods tailored to volumetric data and approaches specific to prostate MRI.

### 2.1. GAN-Based Generative Models for 3D Medical Image Synthesis

Initial attempts to extend GANs to three-dimensional data included the 3D Auto-Encoding GAN [15], based on the α-GAN framework, which demonstrated the feasibility of volumetric MRI synthesis. Jung et al. [16] introduced a hybrid 2D/3D GAN that used a 2D generator to synthesize individual slices and a 3D discriminator to enforce anatomical coherence across the volume, an approach designed for Alzheimer’s brain MRI. While this hybrid design reduces computational complexity, the reliance on slice-wise generation prevents the model from learning a fully volumetric generative representation, thereby limiting intrinsic 3D anatomical consistency and scalability to high-resolution volumetric synthesis.

To overcome GPU memory constraints when generating high-resolution 3D images, HA-GAN [17] proposed a hierarchical framework that synthesizes a coarse low-resolution volume and subsequently refines it using high-resolution patches. Although this strategy effectively reduces memory requirements, the global anatomical structure is fixed at a coarse scale, with fine details introduced only through local patch refinement. As a result, high-resolution anatomical coherence is not modelled end-to-end at the volumetric level, which may restrict volumetric consistency across slices.

3D-StyleGAN [18] adapted the StyleGAN2 architecture to volumetric medical data by modifying components such as filter depth and latent space size. The model achieved high-quality synthesis of 3D brain MRI and provides a strong baseline for volumetric generation. This model is therefore used as a baseline in the experiments to benchmark the performance of the 3D-StyleGAN2-ADA adaptation for T2W MRI synthesis.

Recent GAN-based advances in medical imaging have increasingly focused on cross-modality translation rather than unconditional volumetric generation. For example, Deformation-aware GAN (DA-GAN) [19] addresses synthesis under substantial spatial misalignment by jointly modelling deformation and image translation. Multi-resolution Guided 3D GANs [20] introduce explicit multi-scale feature alignment to improve structural consistency in volumetric translation tasks. Similarly, cross-modality GAN frameworks for CT/MRI synthesis [21] leverage adversarial learning to enhance segmentation performance through modality conversion. While these approaches demonstrate the effectiveness of GANs for conditional image translation, they are inherently designed for modality conversion and therefore rely on an input modality.

### 2.2. Prostate MRI Synthesis

Although synthetic data generation for prostate imaging has been explored, most remain constrained to 2D settings. ProstateGAN [22] trained a conditional GAN to generate 32×32 DWI patches based on Gleason score labels, enabling lesion-specific texture generation. A semi-supervised method for biparametric MRI synthesis [23] introduced an adversarial autoencoder with a custom StitchLayer to produce paired Apparent Diffusion Coefficient (ADC) and T2W slices, followed by cross-modality translation.

Yang et al. [24] proposed a semi-supervised sequential GAN framework for paired slice-wise T2W and ADC synthesis, where two generative networks are trained in sequence to model cross-modality relationships. The method alternates between supervised learning on paired images using reconstruction losses and unsupervised adversarial training to enhance realism and diversity.

Overall, prior work has established important foundations for volumetric GAN-based synthesis. However, existing approaches are often limited by low spatial resolution, hybrid slice-wise designs, hierarchical coarse-to-fine generation, or a primary focus on cross-modality translation. As a result, fully volumetric high-resolution synthesis that models anatomical structure end-to-end remains comparatively underexplored, particularly for small organs such as the prostate.

## 3. Materials and Methods

### 3.1. Dataset

The dataset used to train the models was derived from the publicly available PiCAI (Prostate Cancer AI Challenge) dataset [25], a large-scale multi-centre prostate MRI collection. It contains 1500 anonymized prostate bpMRI scans from 1476 patients, of which 425 are labelled as csPCa and 1075 as indolent or non-significant. Class labels are provided as part of the dataset annotations.

The T2W sequences were used for model training at three spatial resolutions: 64×64×24, 128×128×24, and 256×256×24. Due to inter-scan variability in in-plane field of view (FOV) across centres, scans with markedly larger physical coverage were centre-cropped based on their real-world spatial dimensions (image size and voxel spacing) to match the typical FOV observed across the dataset. All volumes were subsequently resampled to a uniform voxel spacing of 0.5×0.5×3.0 mm and then resized to the target input resolutions. Finally, voxel intensities were normalized to the range [−1,1]. All inputs to the model therefore consist of normalized 3D volumes with a single intensity channel.

### 3.2. 3D-StyleGAN2-ADA Architecture

StyleGAN2 [26] is a style-based GAN architecture that generates high-fidelity images using an intermediate latent space and progressive synthesis. A latent code z∈Z is mapped to a style vector w=m(z)∈W via a learned multilayer perceptron, which modulates the generator through adaptive instance normalization (AdaIN), allowing control over the appearance of generated images. The generator begins from a learned constant and progressively upsamples features using modulated convolutions, with injected noise promoting stochastic variation. When trained conditionally, class labels are embedded using a learned lookup table and integrated into the mapping network. The discriminator mirrors this structure with progressive downsampling and projection-based conditioning.

To address the overfitting risk associated with small datasets, StyleGAN2-ADA introduced Adaptive Discriminator Augmentation (ADA), which applies input augmentations to the discriminator. The augmentation strength is automatically adjusted during training based on discriminator feedback, increasing when overfitting is detected. This helps maintain training stability and generalization, especially when labelled data are limited.

**Figure 1:**
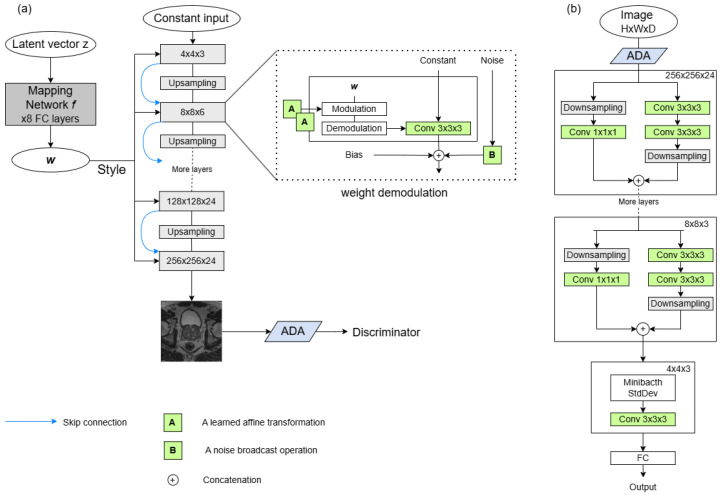
Architecture of StyleGAN2-ADA. (**a**) Generator and (**b**) Discriminator, adapted for 3D volumetric synthesis.

In this work, StyleGAN2-ADA is adapted to enable 3D volumetric synthesis of prostate MRI. All 2D operations, including convolutions, upsampling, and noise injection, are replaced with their 3D counterparts. The overall architecture, training objectives, regularization mechanisms, and support for both unconditional and label-conditioned synthesis are preserved, ensuring architectural consistency with the original formulation. Figure 1 presents the generator and discriminator architectures adapted for volumetric synthesis.

Because prostate MRI volumes exhibit pronounced anisotropy, with substantially lower through-plane resolution and fewer slices relative to the in-plane dimensions (e.g., 384×384×21), the progressive resolution hierarchy was modified to control downsampling along the depth axis. Specifically, depth transitions across resolutions are derived from the input volumetric depth, and depth reduction is applied only when consistent with this computed schedule. This prevents the depth dimension from collapsing prematurely and preserves true volumetric feature representations throughout the network. The generator mirrors the discriminator’s depth transitions during upsampling, ensuring symmetric volumetric scaling across the network. In addition, the architecture was extended to support variable depth sizes, enabling flexibility across volumetric datasets.

Finally, ADA was also adapted to handle 3D volumes using a slice-wise strategy: each volume of shape (B,C,H,W,D) is temporarily reshaped into (B×D,C,H,W), effectively stacking axial slices along the batch axis. Standard 2D augmentations, such as flipping, rotation, and intensity jitter, are applied identically across all slices, and the result is reshaped back into volumetric form. Importantly, augmentation parameters are sampled per volume rather than per slice. The transforms are then applied identically to all slices belonging to the same volume before reshaping the tensor back to its original shape (B,C,H,W,D). This approach leverages efficient 2D augmentation libraries while preserving spatial coherence across slices.

### 3.3. Training Configurations

The 3D-StyleGAN2-ADA models were trained using the official PyTorch (v1.9.1) implementation of StyleGAN2-ADA, adapted for volumetric data. Training was performed on NVIDIA GPUs with varying memory capacities depending on the target resolution and batch size.

Experiments were conducted at three spatial resolutions: 64×64×24 (Low-Res), 128×128×24 (Mid-Res), and 256×256×24 (High-Res and High-Res-cond). Lower resolutions were used to assess convergence behaviour and anatomical consistency before scaling to the highest-resolution setting.

Unconditional models were trained on 1474 T2W volumes from the PiCAI dataset [25], excluding 26 scans with severe spatial misalignment. Minor misalignments were tolerated to preserve anatomical variability. The conditional model, trained only at the highest resolution (High-Res-cond), used all 1500 volumes with csPCa labels for conditioning.

The StyleGAN2-ADA stylegan2 configuration (Configuration F) [26] was employed, known for its effectiveness in image synthesis. Training hyperparameters were held constant across experiments to isolate the effects of architecture and resolution. Training progress is reported in Kimg (1 Kimg = 1000 real images shown to the discriminator), following the standard convention in StyleGAN-based models.

At the highest resolution (256×256×24), the proposed 3D-StyleGAN2-ADA model has approximately 160 M parameters across the generator and discriminator. Training at this resolution was conducted on four NVIDIA A40 GPUs (48 GB each) using a batch size of 8, achieving a training speed of approximately 10 Kimg per 9 h.

For comparative evaluation, a 3D-StyleGAN baseline [18] was trained using the same preprocessed dataset. This baseline follows the original StyleGAN-v1 architectural principles adapted to volumetric data, employing fixed-depth feature maps and a 3D ResNet discriminator, without adaptive channel scaling or discriminator augmentation.

Both architectures were evaluated across all three spatial resolutions using standard generative fidelity and diversity metrics. Downstream segmentation and radiomic analyses were conducted using the highest-resolution ADA-based model.

Full details on hyperparameters and baseline configurations are provided in Appendix A. In addition, a concise summary of the prior 2D slice-level experiments comparing StyleGAN2-ADA and LDM, which motivated this 3D extension, is provided in Appendix B.

### 3.4. Evaluation Metrics

Model performance was evaluated using three standard metrics for generative models: Fréchet Inception Distance (FID) [27], Kernel Inception Distance (KID) [28], and Generation-related Precision and Recall [29]. These metrics assess fidelity and diversity of generated images and are widely adopted for benchmarking generative quality [30].

- FID [27]: This measures the distance between real and generated feature distributions, assuming they follow multivariate Gaussians. It is computed as:
  $$\mathrm{FID}=∥\mu_{r}-\mu_{g}{∥}^{2}+\mathrm{Tr}\left(\Sigma_{r}+\Sigma_{g}-2\sqrt{\Sigma_{r}\Sigma_{g}}\right),$$
  where μ and Σ are the means and covariances of the features. Lower values indicate better alignment.
- KID [28]: This computes the squared Maximum Mean Discrepancy (MMD^2^) between real and generated features, using a polynomial kernel:
  $$\mathrm{KID}={\mathrm{MMD}}^{2}\left(X,Y\right)=E\left[k\left(x,x^{\prime }\right)\right]+E\left[k\left(y,y^{\prime }\right)\right]-2E\left[k\left(x,y\right)\right].$$
  Unlike FID, KID does not assume Gaussianity and provides an unbiased estimate, making it more reliable for small sample sizes.
- Precision and Recall [29]: These provide complementary views of the quality and diversity of the generated images. Given sets of real (R) and generated (G) samples embedded in a feature space by f(·), these metrics are defined as:
  $$\mathrm{Precision}=\frac{\left|\{\;g\in G|\exists \;r\in R,\;∥f(g)-f(r)∥<\epsilon \;\}\right|}{\left|G\right|},$$
  $$\mathrm{Recall}=\frac{\left|\{\;r\in R|\exists \;g\in G,\;∥f(r)-f(g)∥<\epsilon \;\}\right|}{\left|R\right|}.$$
  High Precision indicates that generated samples lie within the manifold of real data (fidelity), whereas high Recall reflects broad coverage of the real data distribution (diversity). This pair of metrics is particularly useful for detecting imbalances such as mode collapse or incomplete coverage. Throughout this work, they are referred to as Generation-related Precision ($P_{GR}$) and Generation-related Recall ($R_{GR}$) to distinguish them from similarly named metrics used in segmentation contexts.

To account for the volumetric nature of the data, these metrics were computed using two complementary strategies: in the Middle Slice approach, only the central axial slice from each 3D volume was used to evaluate feature quality. In the All Slices approach, the 24 axial slices were concatenated along the batch dimension so that the metrics were computed over the entire volume rather than a single representative slice. Each model was evaluated using 3000 synthetic and 1500 real volumes under both strategies.

### 3.5. Segmentation Experiment and Evaluation

To evaluate the clinical value of synthetic images, a downstream prostate segmentation study was conducted using the nnUNetv2 framework [31], a self-configuring pipeline that adapts architecture, training, and augmentation strategies to the target dataset.

The segmentation model was trained on T2W images from two public datasets: ProstateX [32] and Prostate158 [33]. The combined training set comprised 343 volumes (204 from ProstateX, 139 from Prostate158), following the original training splits. The remaining cases from each dataset formed the test set, comprising 142 ProstateX and 19 Prostate158 volumes.

To investigate the impact of synthetic data, the baseline model was fine-tuned by adding 750 synthetic T2W volumes generated by the unconditional High-Res 3D-StyleGAN2-ADA model. Fine-tuning ran for 150 epochs using the combined set of real and synthetic images. A reduced learning rate of $1\times 10^{-3}$ (vs. the default $1\times 10^{-2}$) was used to enable gradual adaptation while preserving previously learned anatomical representations.

Segmentation performance was evaluated using standard volumetric metrics for the Central + Transition Zones (Class 1) and the Peripheral Zone (Class 2), grouped as follows:

- Overlap-based: Dice coefficient and Intersection over Union (IoU), measuring spatial agreement between predicted and reference masks.
- Boundary-based: Average Surface Distance (ASD) and 95th-percentile Hausdorff Distance (HD95), assessing contour alignment and boundary outliers.
- Classification-based: Precision and Recall, quantifying voxel-wise positive prediction accuracy and sensitivity.

The mathematical definitions of these metrics are provided in Appendix E.

### 3.6. Radiomic Feature Extraction and Evaluation

The anatomical plausibility and structural fidelity of the synthetic T2W volumes was evaluated by conducting a radiomic feature analysis using the open-source PyRadiomics library. This analysis aimed to compare the radiomic profiles of synthetic and real images, assessing their alignment across intensity, texture, and shape descriptors.

The analysis was performed across four groups of T2W images:

- PiCAI Subset 1: 750 real T2W images from the PiCAI dataset, used as the primary reference group.
- PiCAI Subset 2: 750 independently sampled real T2W images from PiCAI, used to estimate natural variability across clinical sites and acquisition protocols.
- Synthetic High-Res: 750 images generated by the unconditional 3D-StyleGAN2-ADA model at 256×256×24 resolution.
- Synthetic High-Res-cond: 750 images generated by the label-conditioned version of the model at the same resolution.

All groups, except for the unconditional model, were label-balanced with respect to csPCa class to ensure comparability.

Radiomic features were extracted following the IBSI recommendations [34]. All images and masks were resampled to a uniform voxel spacing, masks were binarized, and anatomical alignment between images and segmentations was verified. Two regions of interest were considered: (i) a fixed prostate-centered bounding box used for first-order and texture features, providing reproducible context across subjects; and (ii) the prostate segmentation mask, used for computing shape descriptors. In total, 130 radiomic features were initially computed per image. Segmentations for real images were obtained from the PiCAI challenge [35], while those for synthetic images were generated with a pretrained nnU-Net trained on real T2W volumes.

Following feature computation, a two-step selection procedure was applied. First, semantic filtering removed redundant or clinically irrelevant descriptors. Second, robustness filtering excluded features with high variability (coefficient of variation > 10%) or strong inter-feature correlation (|ρ|>0.9). The final curated subset comprised 23 features spanning three categories: first-order statistics (Mean, Standard Deviation, Entropy, Skewness, Kurtosis), shape descriptors (Mesh Volume, Surface Area, Sphericity, Elongation, and Maximum 3D diameter), and texture features derived from gray-level matrices (GLCM, GLRLM, GLSZM, GLDM, NGTDM). A detailed description of each feature family can be found in the PyRadiomics documentation [36].

Radiomic similarity between groups was then quantified using standard univariate and multivariate statistics. Per-feature comparisons included QQ-plot alignment ($R^{2}$), Kolmogorov–Smirnov and Levene’s tests, Wasserstein distance, and Spearman correlation; joint behaviour was summarized via Mahalanobis distance, Hotelling’s $T^{2}$, and correlation-matrix dissimilarity (Frobenius norm). To aid interpretation, global structure was visualized with PCA, UMAP, and t-SNE, enabling a concise yet comprehensive assessment of alignment between real and synthetic radiomic profiles.

This evaluation pipeline provided a robust basis for identifying structural inconsistencies and assessing both fidelity and diversity of the synthetic volumes in a clinically meaningful context.

## 4. Results

This section presents the evaluation of 3D-StyleGAN2-ADA models trained at 256×256×24 resolution, compared against a baseline 3D-StyleGAN implementation [18]. High-Res and High-Res-cond refer to the unconditional and class-conditioned ADA-based models, respectively, while High-Res-baseline denotes the non-ADA baseline. Image-level synthesis metrics, a class-wise analysis of dataset imbalance, and validation of synthetic image realism are reported through downstream segmentation and radiomic experiments. Supplementary experiments at lower resolutions are detailed in Appendix C, with additional qualitative results provided in Appendix D.

### 4.1. Quantitative Evaluation Results

Table 1 summarizes the quantitative performance of the models trained at 256×256×24 resolution. Both ADA-based variants (unconditional and conditional) achieved considerably better results than the baseline, with lower FID and KID and higher $P_{GR}$, particularly when evaluated across all slices. These results indicate that both models are able to generate images with high fidelity and strong alignment with the real data distribution.

In contrast, the High-Res baseline failed to converge at 256×256×24, as reflected by substantially higher FID and KID values and lower $P_{GR}$ and $R_{GR}$, with $R_{GR}$ dropping to zero when evaluated on middle slices. This behavior indicates that the 3D StyleGAN baseline was unable to learn a stable representation of the high-resolution volumetric data under the tested configuration. In comparison, the proposed 3D-StyleGAN2-ADA adaptation achieved stable training and consistent generative performance at the same target resolution. While the present study does not isolate the individual contributions of architectural refinements, regularization strategies, and adaptive augmentation, the empirical results demonstrate that the combined framework provides a more robust basis for high-resolution volumetric MRI synthesis.

**Figure 2:**
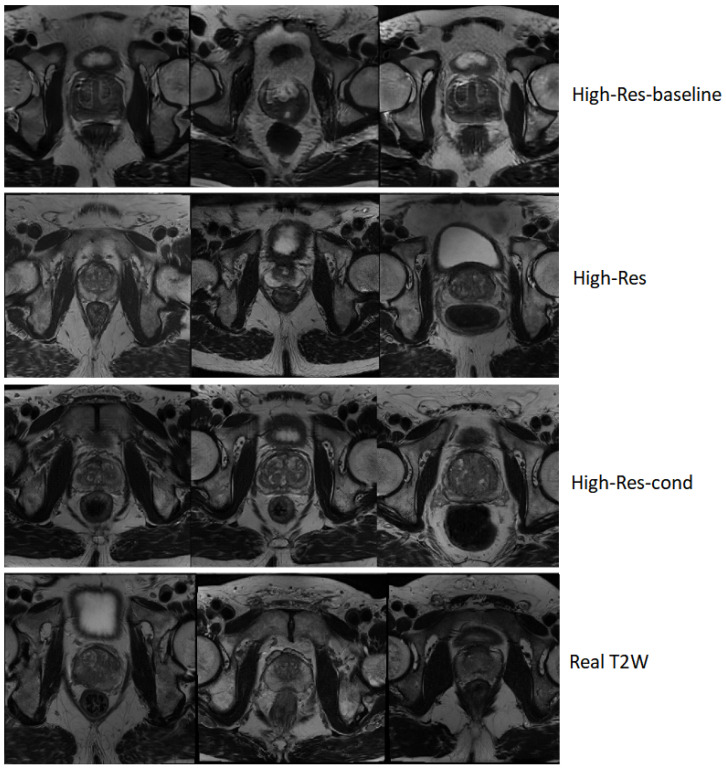
Qualitative comparison of axial T2W slices generated by the models at 256×256×24 resolution. The baseline model images exhibit anatomically inconsistent gland structures and irregular texture patterns, whereas both ADA-based variants produce anatomically coherent prostate morphology and realistic soft-tissue contrast comparable to real data.

A qualitative comparison of samples generated by the three models is presented in Figure 2. The figure contrasts representative axial slices from the baseline and the ADA-based models against real T2W volumes. The High-Res-baseline samples exhibit visibly unrealistic anatomical structures, irregular gland boundaries, and inconsistent internal texture patterns that deviate from real prostate morphology. In contrast, both ADA-based models generate anatomically coherent gland shapes, plausible zonal structure, and realistic soft-tissue contrast. Differences between the unconditional and conditional variants are minimal in terms of perceptual image quality, consistent with the quantitative metrics.

While both ADA-based models achieved high fidelity, the conditional variant yielded lower $R_{GR}$, likely reflecting the difficulty of capturing the broader distribution of appearances within the csPCa class under class-conditioning. The pronounced class imbalance in the training data ( 72% negative cases) may have further biased the generator toward the dominant class.

To explore potential class imbalance effects, a stratified evaluation using 1500 synthetic T2W volumes per model was conducted. For the High-Res-cond variant, 1000 samples were conditioned on non-csPCa and 500 on csPCa, matching the real class ratio. Each set was compared separately to real reference subsets: all PiCAI cases, non-csPCa, and csPCa (Table 2). When evaluated against non-csPCa references, both models showed better alignment across FID, KID, and $P_{GR}$. Comparisons to csPCa subsets yielded consistently higher FID/KID and slightly lower $R_{GR}$, suggesting that the synthetic samples may more closely resemble the dominant class. While these differences are modest, their consistent direction across metrics suggests that modelling the diversity within csPCa appearances remains more challenging, warranting further investigation with minority-aware training strategies.

### 4.2. Downstream Segmentation Performance

The segmentation performance obtained after fine-tuning the nnUNetv2 model with 750 synthetic T2W images is presented in Table 3. Reported values are weighted averages across the ProstateX and Prostate158 test sets, presented separately for the Central + Transition Zone (Class 1) and Peripheral Zone (Class 2).

**Figure 3:**
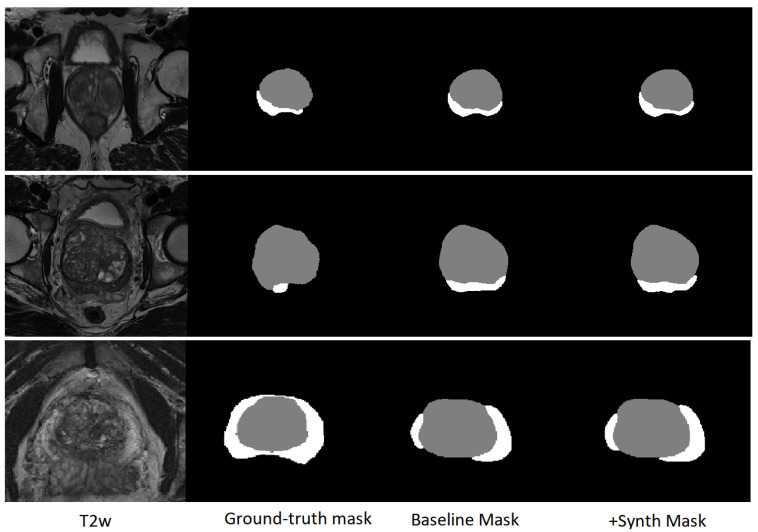
Representative segmentation predictions on test cases. From left to right: T2W image, ground-truth mask, baseline nnU-Net v2 prediction (trained on real data only), and prediction after fine-tuning with 750 synthetic T2W images (+Synth).

Incorporating synthetic data yielded segmentation performance statistically indistinguishable from the baseline model trained on real data alone. Preservation of performance across both anatomical regions supports that volumetric generation maintains structural integrity and anatomically relevant boundaries. Representative segmentation predictions are shown in Figure 3, together with the corresponding T2W images and ground-truth masks. Visually, the delineations produced by the baseline and +Synth models are highly consistent across cases, with only minor local differences (e.g., slightly improved peripheral zone coverage in the bottom example), in agreement with the quantitative results. These findings provide task-based validation that the synthetic images preserve segmentation-relevant features consistent with real prostate MRI.

### 4.3. Radiomic Evaluation of Synthetic Data

Radiomic features (23 total; see Section 3.6) were analyzed to quantify the realism of synthetic T2W images with respect to the real PiCAI Subset 1 (reference). Three pairwise comparisons were conducted: PiCAI Subset 2 vs. reference (real–real), High-Res (unconditional) vs. reference, and High-Res-cond vs. reference.

Univariate comparisons showed partial preservation of radiomic distributions in synthetic images. Several features, including entropy, elongation, and various textural descriptors, displayed good agreement with real data across Levene, Kolmogorov–Smirnov, and Wasserstein tests, comparable to the real–real baseline. However, both synthetic models exhibited notable discrepancies in volume, surface area, and shape emphasis metrics, with significantly shifted variances and distribution shapes. These deviations were more frequent and pronounced than in the real–real comparison, suggesting that while typical anatomical appearances are well captured, the range of radiomic variability, particularly for spatial and shape-based features, may be underrepresented.

Multivariate evaluation complemented the univariate analysis by focusing on global feature structure rather than individual descriptors. Both models achieved very high quantile alignment (QQPlot $R^{2}$ > 0.99) and low Mahalanobis distances, indicating close alignment in multivariate feature space. Hotelling’s T^2^ tests yielded no statistically significant differences (p>0.8), while average correlation similarity remained high and Frobenius distances (0.68–0.79) suggested mild but acceptable deviations in inter-feature structure. These results confirm that synthetic images preserve global radiomic relationships with fidelity comparable to the real–real subset comparison.

**Figure 4:**
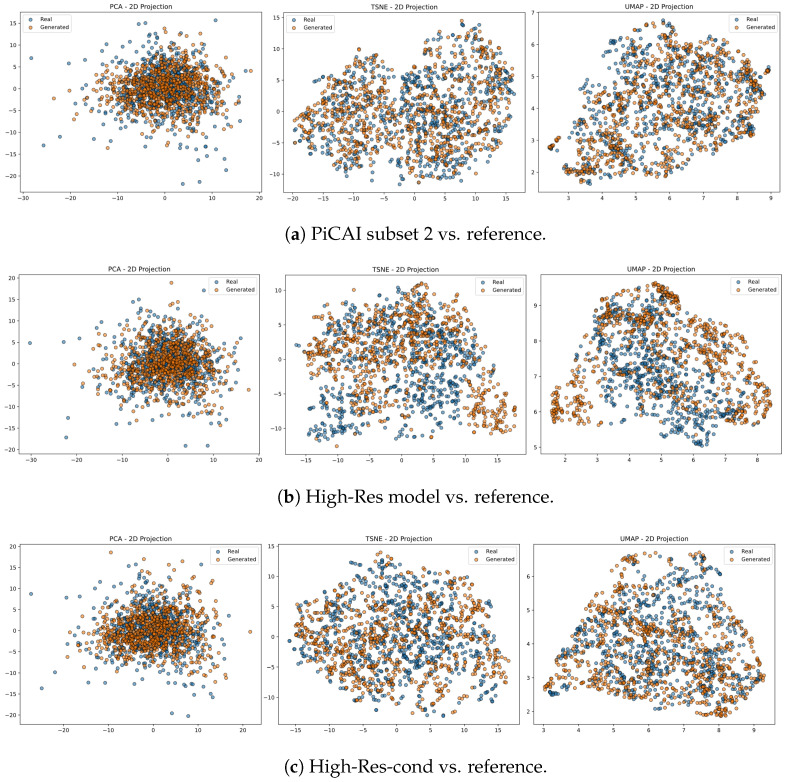
2D projections of radiomic feature space for real and synthetic datasets. (**a**) PiCAI subset 2 vs. reference dataset, shown using PCA (left), t-SNE (middle), and UMAP (right) projections. (**b**) High-Res model vs. reference dataset. (**c**) High-Res-cond model vs. reference dataset.

Figure 4 illustrates this alignment via PCA, t-SNE, and UMAP projections of the radiomic space. Real and synthetic samples show substantial spatial overlap, with only minor distributional shifts. Complete numerical results for the multivariate analysis are summarized in Table 4.

## 5. Discussion

The volumetric extension of StyleGAN2-ADA was explored to determine whether its generative capabilities can be leveraged for 3D prostate MRI synthesis. Prior slice-level experiments [14] showed that StyleGAN2-ADA achieved higher fidelity and substantially faster training and inference than an LDM, motivating its selection for volumetric modelling. Unlike previously reported 3D GAN-based approaches that rely on hybrid slice-wise generation or hierarchical coarse-to-fine strategies, the adapted architecture operates end-to-end in 3D. In the experiments, the model achieved stable training and anatomically coherent volumetric outputs at a target resolution of 256×256×24, whereas the 3D StyleGAN baseline was unable to learn a stable representation of the high-resolution volumetric data. These findings indicate that the StyleGAN2-ADA framework, together with the proposed volumetric adaptations, provides a stable and effective basis for high-resolution 3D prostate MRI synthesis. Disentangling the individual contributions of architectural refinements, regularization strategies, and adaptive augmentation remains an important direction for future work.

Beyond achieving convergence at the targeted resolution, the model produced synthetic images that maintained downstream segmentation performance. Fine-tuning a segmentation network with synthetic data resulted in performance statistically comparable to real-data training across anatomical regions, including the structurally complex peripheral zone. The results support the structural fidelity and practical compatibility of 3D-StyleGAN2-ADA synthetic data within downstream analysis pipelines.

Radiomic evaluation provided complementary evidence of realism. In multivariate analyses, both synthetic models preserved global feature structure, with high feature correlation similarity and limited deviation in inter-feature relationships. These results indicate that the overall radiomic space is well approximated at a global level.

However, univariate tests revealed broader feature-level deviations in synthetic–real comparisons than in the real–real baseline. Discrepancies in spatial and shape-related metrics such as volume, surface area, and large area emphasis suggest that the range of radiomic variability may be underrepresented, a finding aligned with lower recall scores and evidence of class imbalance. Accordingly, further refinement of volumetric synthesis, particularly with respect to diversity, remains an important direction for future work, especially to support the incorporation of synthetic data into downstream deep learning tasks such as prostate cancer detection.

In addition to diversity limitations, the model generated volumes that contained non-realistic structures or anatomical boundaries that do not align with real prostate MRI. Although these cases represented a very small fraction of the generated samples, they highlight a known risk of GAN-based medical image synthesis, namely the potential generation of incorrect or hallucinated content. To mitigate this issue, a lightweight quality-control step based on slice-level structural similarity to real images was applied during generation, discarding samples below a predefined threshold. While effective at filtering evident failure cases, more systematic validation strategies would be advisable if synthetic data are to be used in more sensitive downstream settings, in order to further minimize the inclusion of anatomically implausible samples.

In summary, 3D-StyleGAN2-ADA enables stable high-resolution MRI synthesis with strong anatomical fidelity, integrates into downstream segmentation workflows without performance degradation, and maintains overall radiomic structure. Nevertheless, limitations in diversity, particularly regarding size, shape, and class-specific features, remain important challenges. Future work will focus on investigating methods to better address class imbalance and improve minority-class diversity in volumetric prostate MRI synthesis.

## 6. Conclusions

This work examined whether a volumetric extension of StyleGAN2-ADA can support stable and clinically relevant 3D prostate T2W MRI synthesis. The results demonstrate that this 3D adaptation produces volumetrically coherent samples that show improved distributional fidelity according to FID, KID, and generative Precision–Recall metrics, and that can support downstream segmentation performance. Radiomic analysis further indicated strong global feature alignment between real and synthetic volumes. At the same time, the analysis underscores challenges related to class imbalance and modelling heterogeneous csPCa appearances, indicating that further work is needed to improve minority-class representation and diversity.

## Figures and Tables

**Table 1:** Comparison of 3D-StyleGAN2 and 3D-StyleGAN2-ADA for the 256×256×24 resolution using middle and all 1/1 slices. Arrows indicate the direction of better performance (↓ lower is better; ↑ higher is better).

Model	Slice Type	FID ↓	KID ($\times 10^{-3}$) ↓	$P_{\mathrm{GR}}$ ↑	$R_{\mathrm{GR}}$ ↑
High-Res-baseline	Middle	114.202	91.938	0.216	0.000
	All	8.668	1.110	0.494	0.034
High-Res	Middle	27.260	9.670	0.821	0.205
	All	0.700	0.092	0.584	0.410
High-Res-cond	Middle	36.670	16.260	0.803	0.044
	All	1.470	0.244	0.647	0.202

**Table 2:** Class-wise middle-slice metrics for the High-Res and High-Res-cond models. Arrows indicate the direction of better performance (↓ lower is better; ↑ higher is better).

Model	Reference Set	FID ↓	KID ↓	$P_{\mathrm{GR}}$ ↑	$R_{\mathrm{GR}}$ ↑
High-Res	All cases	36.247	0.017	0.878	0.099
	non-csPCa cases	38.680	0.018	0.883	0.103
	csPCa cases	57.606	0.019	0.912	0.087
High-Res-cond	All cases	46.737	0.026	0.783	0.031
	non-csPCa cases	48.194	0.025	0.783	0.032
	csPCa cases	68.861	0.030	0.805	0.029

**Table 3:** Class-wise segmentation performance across both test sets (ProstateX and Prostate158). Values are weighted averages per class. CTZ denotes the Central + Transition Zone and PZ the Peripheral Zone. Baseline refers to training on real data only; +Synth indicates fine-tuning with 750 synthetic T2W images. Best scores per row and zone are shown in bold. Arrows indicate the direction of better performance (↓ lower is better; ↑ higher is better).

Metric	CTZ Baseline	CTZ +Synth	PZ Baseline	PZ +Synth
Dice ↑	0.9101	**0.9104**	0.8291	**0.8310**
ASD ↓	0.6034	**0.5957**	0.7167	**0.7039**
HD95 ↓	8.956	**8.763**	13.17	**13.16**
IoU ↑	0.8340	**0.8346**	0.7066	**0.7087**
Precision ↑	**0.9195**	0.9176	**0.8342**	0.8328
Recall ↑	0.8925	**0.8947**	0.7847	**0.7926**

**Table 4:** Multivariate radiomic comparison using PCA-reduced feature space. Metrics include distributional similarity (QQPlot R^2^, Mahalanobis distance), structure alignment (Hotelling’s T^2^ and *p*-value, Frobenius), and feature-wise agreement (correlation similarity).

Comparison	QQPlot R^2^	Mahalanobis	T^2^	*p*-Value	Frobenius	Corr. Sim.
PiCAI Subset 2 vs. reference	0.988	0.203	12.104	0.365	0.390	0.955
High-Res vs. reference	0.990	0.160	7.426	0.833	0.678	0.913
High-Res-Cond vs. reference	0.991	0.150	6.841	0.816	0.792	0.894

**Figure A1:**
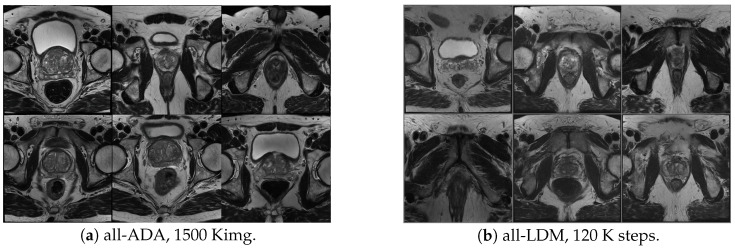
2D slice-level qualitative comparison. (**a**) Samples generated by StyleGAN2-ADA (all-ADA model) after 1500 kimg of training. (**b**) Samples generated by the LDM model (all-LDM model) after 120 K training steps. StyleGAN2-ADA produces sharper gland boundaries and more coherent textures than the LDM.

**Figure A2:**
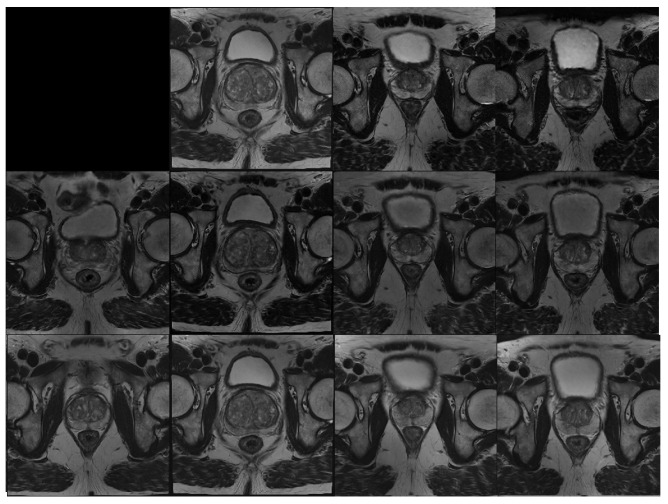
Style mixing example. Middle slices from synthetic volumes are shown. The first row and first column correspond to images generated from independent latent codes. Each remaining image combines anatomical structure from the column source (coarse styles) with intensity and contrast features from the row source (fine styles), demonstrating disentangled control over structure and appearance.

## Data Availability

The data presented in this study are openly available in PiCAI (Prostate Cancer AI Challenge) at https://pi-cai.grand-challenge.org/ (accessed on 19 May 2023). The dataset is described in detail in [25].

## Abbreviations

The following abbreviations are used in this manuscript:

ADA	Adaptive Discriminator Augmentation
ADC	Apparent Diffusion Coefficient
ASD	Average Surface Distance
bpMRI	Biparametric Magnetic Resonance Imaging
CNN	Convolutional Neural Network
csPCa	Clinically Significant Prostate Cancer
Dice	Dice Similarity Coefficient
DL	Deep Learning
DWI	Diffusion-Weighted Imaging
FID	Fréchet Inception Distance
FOV	Field of View
GAN	Generative Adversarial Network
GLCM	Gray-Level Co-occurrence Matrix
GLDM	Gray-Level Dependence Matrix
GLRLM	Gray-Level Run-Length Matrix
GLSZM	Gray-Level Size Zone Matrix
GPU	Graphics Processing Unit
HD	Hausdorff Distance
IBSI	Imaging Biomarker Standardisation Initiative
IoU	Intersection over Union
KID	Kernel Inception Distance
Kimg	Kilo-Images (1000 real images seen by discriminator)
ML	Machine Learning
MRI	Magnetic Resonance Imaging
NGTDM	Neighboring Gray-Tone Difference Matrix
PCa	Prostate Cancer
PCA	Principal Component Analysis
PiCAI	Prostate Cancer AI Challenge
$P_{GR}$	Generation-related Precision
$R_{GR}$	Generation-related Recall
T2W	T2-weighted (MRI sequence)
UMAP	Uniform Manifold Approximation and Projection

## Appendix A. Training Resources and Configurations

This section summarizes the hyperparameters and baseline configurations used for training the models.

### Appendix A.1. Training Hyperparameters

The main training hyperparameters used across all 3D-StyleGAN2-ADA models are summarized below. These were kept constant across experiments to isolate the effects of architecture and resolution.

- Latent space: 512-dimensional latent vector ($z\in R^{512}$) mapped to an intermediate latent space ($w\in R^{512}$) via an 8-layer MLP.
- Synthesis network: Progressive 3D convolutions with a maximum of 512 channels.
- Loss: Non-saturating GAN loss with R1 regularization (γ=10).
- Optimizer: Adam with learning rate 0.002, $\beta_{1}=0$, $\beta_{2}=0.99$, and $\epsilon =10^{-8}$.
- Augmentation: ADA with $r_{t}=0.6$, including flipping, rotation, scaling, brightness/contrast, hue/saturation shifts, and luma flips.

### Appendix A.2. 3D-StyleGAN Baseline Configuration

The 3D-StyleGAN baseline models were trained using the configuration Gorig-Dres-DeepFil-R1-3d-2mm from the official implementation of Hong et al. [37]. This setup employs the original 3D-StyleGAN generator, which adapts the StyleGAN v1 architecture to volumetric data by replacing all 2D operations with their 3D counterparts. The generator incorporates modulation and demodulation operations and injects spatially adaptive 3D Gaussian noise at each block, preserving the style-based control mechanism. The discriminator follows a custom 3D ResNet architecture that uses the non-saturating logistic loss without gradient penalty regularization. Both generator and discriminator maintain fixed feature map depths per resolution level and do not implement the adaptive channel scaling introduced in StyleGAN2, where feature map depth decreases with increasing spatial resolution.

Filter depth, chosen based on GPU memory constraints, and base feature map sizes for the baseline models are listed below:

- Low-Res-baseline: Depth 512, base size 8×8×3.
- Mid-Res-baseline: Depth 256, base size 16×16×3.
- High-Res-baseline: Depth 128, base size 32×32×3.

## Appendix B. Prior 2D Slice-Level Comparison (StyleGAN2-ADA vs. LDM)

Prior 2D slice-level experiments on PiCAI T2W images are provided next for context. These experiments, in which StyleGAN2-ADA was compared with the LDM, were conducted as part of an earlier study to evaluate performance for prostate 2D T2W image generation [14]. In this setting, the models with tag all- were trained using all slices from each T2W volume as input, yielding a total of 33,672 slices. The pos- and neg- models were trained separately using slices from positive and negative csPCa cases, respectively, comprising 9482 and 24,190 slices. Model -NoAug corresponds to the configuration in which ADA was disabled during training.

The models were trained using official PyTorch implementations. FID, KID, and Precision–Recall metrics were computed using 50 K generated images per checkpoint for StyleGAN2-ADA and 10 K for LDM, limited by inference speed.

Quantitative results are reported in Table A1, showing consistently lower FID and KID and higher $P_{GR}$ for StyleGAN2-ADA across both full-dataset and class-specific training regimes. Compared to the all-NoAug variant, the ADA-based model achieved substantially improved distribution alignment, reducing FID from 15.22 to 10.43 and KID from 0.0106 to 0.0062. While Precision remained comparable between the two configurations, Recall was markedly higher with ADA (0.166 vs. 0.0819), indicating improved coverage of the real data distribution and reduced mode collapse. Similar trends were observed in the class-specific experiments, where ADA consistently outperformed LDM-based alternatives. Qualitative examples illustrating these differences are shown in Figure A1, where StyleGAN2-ADA samples exhibit sharper gland boundaries and more coherent textures compared to those generated by the LDM.

**Table A1:** 2D slice-level comparison on PiCAI T2W. Arrows indicate the direction of better performance (↓ lower is better; ↑ higher is better).

Model	FID ↓	KID ↓	$P_{\mathrm{GR}}$↑	Recall ↑
all-ADA (StyleGAN2-ADA)	10.43	0.0062	0.51	0.166
all-NoAug	15.22	0.0106	0.54	0.0819
all-LDM	20.22	0.0171	0.347	0.136
neg-csPCa-ADA	7.54	0.0050	0.57	0.256
pos-csPCa-ADA	18.09	0.0120	0.49	0.149
neg-csPCa-LDM	22.79	0.0193	0.332	0.098
pos-csPCa-LDM	36.83	0.0360	0.29	0.11

In addition to improved fidelity, StyleGAN2-ADA was significantly more efficient. It typically converged in ∼3 h and could generate 50 K images in under one minute. In contrast, the LDM required ∼9–12 h to reach its best checkpoint and over 16 h to generate 10 K samples on a 16 GB Quadro RTX 5000 GPU. These factors, alongside superior generation quality, motivated the adoption of StyleGAN2-ADA for 3D volumetric synthesis.

## Appendix C. Experiments at Lower Resolutions

Supplementary quantitative results for lower-resolution models (64×64×24 and 128×128×24) are presented below. These results complement the main analysis in Section 4, providing baseline comparisons and convergence trends across resolutions. For completeness, the computational requirements associated with training these lower-resolution models are also reported.

At 64×64×24 resolution (Table A2), the ADA-based model achieved substantially lower FID and KID scores, particularly for the all-slice evaluation, despite being trained for fewer iterations (300 Kimg vs. 544 Kimg for the baseline). It also yielded higher $P_{GR}$ on middle slices, indicating improved sample realism, though slightly reduced $R_{GR}$ suggested a minor trade-off in diversity.

At 128×128×24 resolution (Table A3), 3D-StyleGAN2-ADA again outperformed the baseline across nearly all metrics, achieving lower FID/KID and higher $P_{GR}$ and $R_{GR}$. While the baseline required 528 Kimg to plateau, the ADA model reached superior quality earlier (440 Kimg), demonstrating more sample-efficient training. These results collectively confirm that ADA mechanisms improve both convergence stability and image fidelity at multiple scales.

**Table A2:** Comparison of 3D-StyleGAN2 and 3D-StyleGAN2-ADA at 64×64×24 resolution using middle and all slices. Arrows indicate the direction of better performance (↓ lower is better; ↑ higher is better).

Model	Slice Type	FID ↓	KID ($\times 10^{-3}$) ↓	$P_{\mathrm{GR}}$ ↑	$R_{\mathrm{GR}}$ ↑
Low-Res-baseline	Middle	20.113	7.123	0.745	0.421
	All	5.858	0.306	0.597	0.471
Low-Res	Middle	19.022	4.678	0.783	0.407
	All	1.049	0.212	0.594	0.384

**Table A3:** Comparison of 3D-StyleGAN2 and 3D-StyleGAN2-ADA at 128×128×24 resolution using middle and all slices. Arrows indicate the direction of better performance (↓ lower is better; ↑ higher is better).

Model	Slice Type	FID ↓	KID ($\times 10^{-3}$) ↓	$P_{\mathrm{GR}}$ ↑	$R_{\mathrm{GR}}$ ↑
Mid-Res-baseline	Middle	30.362	16.320	0.786	0.067
	All	3.736	0.174	0.655	0.383
Mid-Res	Middle	19.443	4.070	0.807	0.390
	All	0.703	0.284	0.600	0.418

The 64×64×24 model contains approximately 145 M parameters across generator and discriminator and was trained using four NVIDIA GeForce RTX 2080 Ti GPUs (11 GB) with a batch size of 8. Training progressed at approximately 10 Kimg per 5 h and 40 min, and the model was trained for 340 Kimg, corresponding to roughly 192.5 h of wall-clock time.

The 128×128×24 model increases capacity to approximately 156 M parameters and was trained using four NVIDIA GeForce RTX 3090 GPUs (24 GB) with a batch size of 8. Under this configuration, training required close to 5 h per 10 Kimg and was run for 440 Kimg, corresponding to approximately 219.5 h of wall-clock time.

## Appendix D. Style Mixing

Figure A2 shows the result of style mixing applied using the High-Res model. Each cell in the grid combines style vectors from two independently sampled latent codes. The first row and first column contain reference images generated without mixing. For the remaining cells, coarse (low-resolution) styles are taken from the column latent code, while fine (high-resolution) styles are taken from the row latent. This enables controlled recombination of structural and appearance features.

The results illustrate the model’s capacity to disentangle anatomical layout from intensity-based appearance. Images in the same column retain consistent spatial structures, such as prostate and bladder shape, determined by the coarse styles of the column latent. In contrast, images across rows vary in intensity and contrast, reflecting the fine styles of the row latent. These findings indicate that the High-Res model supports independent control over structural and visual attributes, reinforcing its suitability for controlled medical image synthesis.

## Appendix E. Segmentation Metrics Definitions

Let *P* denote the predicted binary mask and *G* the ground-truth mask.

The Dice coefficient is defined as:

(A1) $$\mathrm{Dice}=\frac{2|P\cap G|}{\left|P|+|G\right|}.$$

The Intersection over Union (IoU) is:

(A2) $$\mathrm{IoU}=\frac{\left|P\cap G\right|}{\left|P\cup G\right|}.$$

Precision and Recall are defined voxel-wise as:

(A3) $$\mathrm{Precision}=\frac{\mathrm{TP}}{\mathrm{TP}+\mathrm{FP}},\;\mathrm{Recall}=\frac{\mathrm{TP}}{\mathrm{TP}+\mathrm{FN}},$$

where TP, FP, and FN denote true positives, false positives, and false negatives.

Let $S_{P}$ and $S_{G}$ denote the surface points of the predicted and ground-truth masks. The Average Surface Distance (ASD) is defined as:

(A4) $$\mathrm{ASD}=\frac{1}{|S_{P}\left|+\right|S_{G}|}\left(\sum_{p\in S_{P}}d\left(p,S_{G}\right)+\sum_{g\in S_{G}}d\left(g,S_{P}\right)\right),$$

where d(x,S) is the minimum Euclidean distance from point *x* to surface *S*.

The 95th-percentile Hausdorff Distance (HD95) is defined as the 95th percentile of the bidirectional surface distance distribution between $S_{P}$ and $S_{G}$, reducing sensitivity to extreme outliers.
